# Biosocial predictors and blood pressure goal attainment among postmenopausal women with hypertension

**DOI:** 10.3389/fcvm.2024.1268791

**Published:** 2024-02-16

**Authors:** Geetha Kandasamy, Thangamani Subramani, Gigi Sam, Mona Almanasef, Tahani Almeleebia, Eman Shorog, Asma M. Alshahrani, Amjad Hmlan, Atheer Y. Al Suhaym, Kousalya Prabahar, Vinoth Prabhu Veeramani, Palanisamy Amirthalingam

**Affiliations:** ^1^ Department of Clinical Pharmacy, College of Pharmacy, King Khalid University, Abha, Saudi Arabia; ^2^ Department of Pharmacy Practice, Grace College of Pharmacy, Palakkad, India; ^3^ Department of Pharmaceutical Sciences, College of Pharmacy, Shaqra University, Dawadmi, Saudi Arabia; ^4^ Eradah Hospital and Mental Health in Jazan, Ministry of Health, Jazan, Saudi Arabia; ^5^ Department of Pharmacy Practice, Faculty of Pharmacy, University of Tabuk, Tabuk, Saudi Arabia

**Keywords:** hypertension, postmenopausal women, BP goal, age at menopause, biosocial predictors

## Abstract

**Objectives:**

In postmenopausal states, women may not maintain blood pressure (BP) in the same way as men, even though most women follow their treatment plans and prescriptions more consistently than men. Biological and lifestyle factors influence the progression of hypertension in postmenopausal women (PMW). This study aimed to determine biosocial predictors associated with achieving the target BP in PMW with hypertension.

**Methods:**

A prospective observational study was conducted in the General Medicine Department at Karuna Medical College Hospital, Kerala, India. The definition of BP goal attainment was established based on the guidelines outlined by the VIII Joint National Committee 2014 (JNC VIII). Multivariate logistic regression analysis was used to analyse biosocial predictors, such as educational status, employment status, body mass index (BMI), number of children, age at menarche, age at menopause, and number of co-morbidities, associated with BP goal achievement.

**Results:**

Of the patients, 56.4% achieved their BP goals on monotherapy and 59.7% achieved it on combination therapy. Level of education [odds ratio (OR) = 1.275, 95% confidence interval (CI): 0.234–7.172], employment status (OR = 0.853, 95% CI: 0.400–1.819), age at menopause (OR = 1.106, 95% CI: 0.881–1.149), number of children (OR = 1.152, 95% CI: 0.771–1.720), BMI (OR = 0.998, 95% CI: 0.929–1.071), and number of co-morbidities (OR = 0.068, 95% CI: 0.088–1.093) did not show a significant relationship, and age at menarche (OR = 1.577, 95% CI: 1.031–2.412) showed a significant association with BP goal attainment among hypertensive postmenopausal women.

**Conclusion:**

Half of the hypertensive postmenopausal women did not achieve their BP goals. Interventions are required to expand screening coverage and, under the direction of medical professionals, there should be plans to improve hypertension control and increase awareness of the condition.

## 1 Introduction

Hypertension is the main cause of cardiovascular (CV) morbidity and mortality, with an estimated impact of more than 10 million deaths annually attributable to high blood pressure (BP). Hypertension is a condition characterised by greater pressure exerted by blood flow on vessel walls than usual. It is frequently referred to as the “silent killer” because many individuals are unaware that they have it (1–3). Poor regulation of blood pressure is a significant risk factor for myocardial infarction, cardiac failure, and stroke, with only 30% of hypertensive individuals having blood pressure within the normal range (4).

According to data on global health, half of all deaths from heart disease and stroke are attributed to hypertension, and one-third of all adults worldwide suffer from this condition (5). Adverse pathophysiologic effects of hypertension, including chronic renal disease, diabetes, left ventricular hypertrophy, diastolic dysfunction, and cardiac failure, are more prevalent in women than in men. Blood pressure and coronary artery disease are the leading causes of death among female Medicare beneficiaries (6).

Despite a higher adherence to treatment plans and medications and more regular BP monitoring among women than men, the control of BP may not be as effective in women as it is in men (7). Increases in blood pressure are a characteristic of ageing in both men and women, but age-related increases are more pronounced in women compared to men, and postmenopausal women are more prone to hypertension (8). Menopause, a natural event linked to women's ageing, is characterised by a reduction in fertility and reproductive capacity, with its main feature being 12 consecutive months of amenorrhoea (9, 10). It is the complete cessation of menstruation resulting from lowered oestrogen production by ovaries and is viewed as a normal stage in women's lives (11). The average age of menopause is 51 years, but it can occur naturally at any age between 40 and 58 years (12).

To prevent an increase in the burden of hypertension associated with Cardiovascular diseases (CVDs) among women, it is crucial to closely examine the connection between hypertension and risk factors associated with menopause. Understanding elevated blood pressure and its causes is becoming increasingly important to improve current medical services (13). Obesity and overweight among postmenopausal women are common and have been reported to be influenced by diet, a lack of physical activity, hormonal changes, age, parity, education, age at menarche, cigarette use, occupation, monthly income, age during the first and last pregnancy, and a high-calorie diet (14). Early menarche is related to a higher body mass index (BMI) and early exposure to high levels of oestrogen, while late menarche is linked to late exposure to low levels of oestrogen, which is associated with blood pressure (15).

However, numerous studies have shown a link between menopause age and hypertension, and late menopause has been associated with an increased risk of obesity and high blood pressure (16). Half of Indian women with hypertension exhibit substantial risk factors, including age, high BMI, abdominal obesity, and diabetes (17). Meanwhile, Caucasian perimenopausal and postmenopausal women who have had at least one child are more likely to develop early hypertension due to parity than nulliparous women, who face a nearly threefold increased risk (18). Women and men may have various social qualities, as well as diverse environments and living situations, which may have different consequences on the development of hypertension. Women with hypertension are often more affected than men by factors such as increased stress in life, anxiety at work, and depression. Other social determinants of health that affect hypertension in men and women differently include marital status and social support (19).

Poor blood pressure control and high healthcare costs are caused by factors such as poor patient self-care, a high prevalence of self-medication, inaccurate hypertension treatment guidelines, and non-adherence to treatment plans and medical regimens. All these factors contribute to inadequate healthcare knowledge, worsening the situation in India (20). Women have been found to exhibit a higher percentage of non-adherence to antihypertensive treatment. Women typically prioritise taking care of their families over their own needs, pay less attention to their health, and may not be as motivated to follow through with chronic illness treatments (21). Although achieving therapeutic lowering of blood pressure can greatly reduce cardiac morbidity and mortality, two-thirds of hypertensive women remain at risk due to persistent uncontrolled blood pressure. It is crucial to recognise particular risk factors that are specific to events in women (22).

The increasing rates of CVD in postmenopausal women demonstrated over recent decades may accelerate the attention paid to the relationship between hypertension with menopause and associated risk factors. Understanding high blood pressure and its causes is becoming increasingly important to improve current medical services. The aim of this study is to explore several biosocial predictors of hypertension and their potential role in the emergence of high blood pressure in postmenopausal women.

## 2 Methodology

### 2.1 Study settings

A prospective observational study was designed to categorise the biosocial predictors for hypertension among hypertensive postmenopausal women at a private hospital in Kerala per their convenience and study desire. Convenience sampling was used to identify eligible participants. However, 381 of the 415 patients who visited the hospital during the study period and met the inclusion criteria participated; this sample size was considered adequate for this study. Participants voluntarily gave their consent after receiving assurances of confidentiality and anonymity.

### 2.2 Study population

Hypertensive women with a history of natural menopause, who were treated for hypertension and who had used the same class of antihypertensive medication for at least 3 months, were included. Patients using hormone replacement therapy or with chronic kidney disease, osteoporosis, stroke, sleep disorder, or a history of hysterectomy were excluded.

Data on demographic characteristics, employment status, and educational status were obtained using a predesigned data entry form. Social variables such as educational level and employment status were categorised. The number of live births for each woman was recorded in parity. Height (cm) and weight (kg) were measured with the patient barefooted and dressed in light clothing. BMI was calculated using the following formula: weight (kg)/height^2^ (m^2^), and classified according to the WHO International Classification of BMI: normal, 18.5–24.99; overweight, 25–30; and obese, <30 (23, 24). Anthropometric measurements, including height and weight, were taken for calculation; participants were tested twice for BP while seated using a mercury sphygmomanometer, and the average of the two readings was obtained. According to the Joint National Committee VII (2003), hypertension can be defined as a systolic blood pressure (SBP) of 140 mmHg or higher and/or a diastolic blood pressure (DBP) of 90 mmHg or higher.

A sphygmomanometer and a stethoscope were used to measure blood pressure to the nearest 2 mmHg. After each participant took a 10-min break, their blood pressure was monitored. Participants were divided into various categories based on their blood pressure readings; blood pressure values of <120 mmHg systolic and <80 mmHg diastolic were considered normal. Participants with systolic blood pressure readings between 80 and 89 mmHg and diastolic blood pressure readings between 120 and 139 mmHg, respectively, were categorised as having prehypertension. Stage-I hypertension was defined as systolic blood pressure between 140 and 159 mmHg and diastolic blood pressure between 90 and 99 mmHg (Joint National Committee VII, 2003), while Stage-II hypertension was defined as systolic blood pressure >160 mmHg and diastolic >100 mmHg (25).

### 2.3 Age at menarche

The retrospective (or recall) approach was used to obtain information about the age of participants at menarche. This method is used when there is no other way to learn about an event that has not occurred recently, despite the method being rather inaccurate (26).

### 2.4 Age at menopause

Information about the age at menopause was collected by asking the participants to recollect their age at the time of their last menstrual period. Almost all study participants could recall the month and year of onset (menarche) and end (menopause) of their menstrual cycle with precision. The age at menopause was determined by using the middle (15th) of the month (26).

### 2.5 Ethical approval

The study was approved by Karuna Medical College Hospital (KMC/IHEC/10/2019), south India.

### 2.6 Statistical analysis

Descriptive statistics were used to describe the demographic characteristics of the study group, comparing patients who achieved their blood pressure goal with those who did not. For categorical variables, the *χ*^2^ test or Fisher's exact test was used to assess differences between groups. All significant demographic characteristics were incorporated into a logistic regression model to determine the adjusted odds ratio (OR) of factors related to the control of BP. All analyses were carried out using IBM SPSS Statistics version 22.0. A *P*-value of 0.05 was considered statistically significant.

## 3 Results

### 3.1 Sociodemographic details

The analysis included 381 hypertensive postmenopausal women based on the inclusion and exclusion criteria. Table 1 illustrates the demographic features of the study's participants who achieved their BP targets and those who did not. Out of the 381 treated hypertensive individuals, only 55% (*n* = 210) achieved their blood pressure targets, while 45% (*n* = 171) failed to achieve their blood pressure goals.

**Table 1:** Demographic characteristics of hypertensive postmenopausal women.

S. No.	Characteristic	*N* (%)			*P*-value
		Total	BP goal achieved	BP goal not achieved	
1	Age group (years)				
	≤50	37 (9.7)	14 (37.8)	23 (62.1)	0.001*
	51–59	190 (49.9)	93 (48.9)	97 (51.1)	
	≥60	154 (40.4)	103 (66.9)	51 (33.1)	
2	Educational status				
	Illiterate	21 (5.5)	16 (76.2)	05 (23.8)	0.001*
	Primary	285 (74.8)	134 (47)	151 (53)	
	Diploma/undergraduate/postgraduate	75 (8.3)	60 (80)	15 (20)	
3	Employment status				
	Employed	86 (22.6)	68 (79.1)	18 (20.9)	0.001*
	Housewife/retired	295 (77.4)	142 (48.1)	153 (51.8)	
4	Age at menopause (years)				
	<50	112 (29.4)	67 (59.8)	45 (40.2)	0.596
	>50	269 (70.6)	153 (56.9)	116 (43.1)	
5	Age at menarche (years)				
	≤15	149 (39.1)	83 (55.7)	66 (44.3)	0.853
	>15	232 (60.9)	127 (54.7)	105 (45.3)	
6	Number of children				
	1–2	226 (59.3)	123 (54)	103 (46)	0.524
	>2	155 (40.7)	87 (56.1)	68 (43.9)	
7	Body mass index (kg/m^2^)				
	Normal weight (18.5–24.9)	96 (25.1)	78 (81.3)	18 (18.7)	0.001*
	Overweight (25–29.9)/obese (30–40)	285 (74.8)	132 (46.3)	153 (53.7)	
8	Number of co-morbidities				
	≤2	278 (72.9)	164 (59)	114 (41)	0.012*
	>2	103 (27)	46 (44.7)	57 (55.3)	

* *P* < 0.05 is considered as significant.

### 3.2 Age group-wise distribution

Hypertensive postmenopausal women aged 51–59 years (*n* = 190) were more frequent than those aged ≤ 50 (n = 37) or ≥ 60 (*n* = 154) years. Those aged ≥ 60 group had a statistically higher number of patients (*P* < 0.01) achieve the BP goal than the other age groups. It was found that increasing age in women was related to BP goal attainment.

### 3.3 Educational status and employment status

Among the patients, those who were illiterate (76%) and those with undergraduate or postgraduate levels of education (80%) showed a significant association with BP goal attainment compared to those with primary-level education. Regarding employment status, employed patients (79.1%) attained the BP target significantly more often compared to patients who were unemployed (48.1%), including those who were housewives or retired. High educational levels and employment status were significantly more associated with reaching the BP target.

### 3.4 Age at menarche, age at menopause, and parity status

Overall, 70.6% of study patients were in the >50 years of age at menopause group, but 59.8% of patients in the <50 years of age at menopause group achieved their BP goals. Also, 60.9% of patients were in the >15 years of age at menarche group, and 55.7% of patients in the ≤15 years of age at menarche group achieved their BP goals. Patients with fewer than two children have shown high levels of BP goal attainment compared to patients with more than two children. Age at menopause, age at menarche, and number of children showed no significant association with BP goal attainment among postmenopausal women with hypertension.

### 3.5 Body mass index

Patients with a normal weight (81.3%) more often attained their BP target than those who were overweight (46.3%), and there was a significant association with BP target attainment. Overweight patients showed low levels of BP target attainment, and a significantly higher number of overweight patients did not achieve their BP goals (*P* < 0.01). Being overweight was significantly associated with not reaching the BP goal.

### 3.6 Number of co-morbidities

Hypertensive postmenopausal women with fewer than two co-morbidities (59%) showed statistically higher levels of BP goal attainment those with more than two co-morbidities (44.7%). The number of co-morbidities was associated with BP goal attainment in the study population.

### 3.7 Type of antihypertensive medication and frequency of BP goal achievement

The class of antihypertensive medication and the frequency of hypertensive postmenopausal women achieving their BP goals are presented in Table 2. Most patients were prescribed at least one antihypertensive medication: 60.9% were treated with monotherapy, and 39.1% were on combination therapy, like two or more antihypertensive medications. Among patients treated with pharmacotherapy, 56.4% (*n* = 131) of patients achieved their BP goals on monotherapy and 59.7% (*n* = 89) achieved their BP goals on combination therapy. In monotherapy, an angiotensin II receptor blocker was prescribed to 35.8%, of which 56.6% achieved their goal; a beta-blocker (BB) was prescribed to 11.6%, of which 59.2% achieved their goal; a calcium channel blocker (CCB) was prescribed to 39.7%, of which 56.5% achieved their goal; and angiotensin-converting enzyme inhibitors (ACEIs) were prescribed to 12.9%, of which 53.3% achieved their goal.

**Table 2:** Types of antihypertensive medications and frequency of achievement of BP goal among hypertensive postmenopausal women.

S. No.	Class of antihypertensive medication	*N* (%)		
		Total	BP target not achieved	BP target achieved
I	Monotherapy (*N* = 232)			
	ACEI	30 (12.9)	14 (46.6)	16 (53.3)
	Angiotensin II receptor blocker (ARB)	83 (35.8)	36 (43.4)	47 (56.6)
	BB	27 (11.6)	11 (44.4)	15 (55.5)
	CCB	92 (39.7)	40 (43.4)	52 (56.5)
II	Combination therapy (*N* = 149)			
	Angiotensin II receptor blocker (ARB) + BB	24 (16)	10 (41.6)	14 (58.3)
	Angiotensin II Receptor blocker (ARB) + CCB	32 (21.5)	13 (40.6)	19 (59.3)
	Angiotensin II receptor blocker (ARB) + diuretics	41 (27.5)	16 (39)	25 (61)
	Angiotensin II receptor blocker (ARB) + BB + diuretics (2)	45 (30.2)	18 (40)	27 (60)
	Angiotensin II receptor blocker (ARB) + CCB + diuretics (2)	07 (4.7)	03 (42.8)	04 (57.2)

In combination therapy, an angiotensin II receptor blocker + beta-blocker was prescribed to 16%, of which 58.3% achieved the goal; an angiotensin II receptor blocker + calcium channel blocker was prescribed to 21.5%, of which 59.3% achieved their goal; an angiotensin II receptor blocker + diuretic (DI) was prescribed to 27.5%, of which 61% achieved their goal; an angiotensin II receptor blocker + beta-blocker + diuretic was prescribed to 30.2%, of which 60% achieved their goal; and an angiotensin II receptor blocker + calcium channel blocker + diuretic was prescribed to 4.7%, of which 57.2% achieved their goal. Approximately 40% of the patients did not achieve their BP goals in both therapies among the study population. An angiotensin II receptor blocker and a calcium channel blocker were commonly prescribed antihypertensive medications in 75% of hypertensive postmenopausal women under monotherapy; this included losartan (35%), telmisartan (65%), amlodipine (78%), and nifedipine (22%). In combination therapy, there were two drugs in the diuretics class prescribed, like loop diuretics combined with potassium-sparing diuretics, in 35% of patients with coronary artery disease.

### 3.8 Factors affecting BP goal attainment in hypertensive postmenopausal women using logistic regression analysis

The association of influencing factors and BP goal attainment among postmenopausal hypertensive women was analysed by logistic regression analysis. Age at menopause, age at menarche, number of children, employment status, level of education, BMI, and number of co-morbidities were used as independent variables, while BP goal attainment was employed as a dependent variable (Table 3). Level of education [OR = 1.297, 95% confidence interval (CI): 0.234–7.172], employment status (OR = 0.853, 95% CI: 0.400–1.819), age at menopause (OR = 1.006, 95% CI: 0.881–1.149), number of children (OR = 1.152, 95% CI: 0.771–1.720), BMI (OR = 0.998, 95% CI: 0.929–1.071), and number of co-morbidities (OR = 0.068, 95% CI: 0.088–1.093) did not show a significant relationship with BP goal attainment. Age at menarche (OR = 1.577, 95% CI: 1.031–2.412) showed a significant association with BP goal attainment among hypertensive postmenopausal women.

**Table 3:** Factors affecting BP goal attainment in hypertensive postmenopausal women using logistic regression analysis.

S. No.	Factors	B	SE	*P*-value	Odds ratio	95% CI
1	Level of education	0.260	0.873	0.766	1.297	0.234–7.172
2	Employment status	−0.160	0.387	0.680	0.853	0.400–1.819
3	Age at menopause	0.006	0.068	0.927	1.006	0.881–1.149
4	Age at menarche	0.455	0.217	0.036*	1.577	1.031–2.412
5	No. of children	0.141	0.205	0.490	1.152	0.771–1.720
6	BMI	−0.002	0.036	0.951	0.998	0.929–1.071
7	Number of co-morbidities	−1.168	0.641	0.068	0.311	0.088–1.093

*P* < 0.05 is considered as significant.

## 4 Discussion

Even though cardiovascular disease is the leading cause of morbidity and mortality in both men and women, women are more likely than men to die from CVD. Increases in blood pressure with age are a hallmark of ageing in both men and women, but they occur earlier in women than in men, and hypertension is a major risk factor for CVD (27). In postmenopausal hypertension, abnormalities in heart size, heart function, and compliance of the heart have been observed and characterised. Compared to young adult women, postmenopausal women have a higher incidence of left ventricular hypertrophy and are more likely to develop diastolic dysfunction (28).

Our study showed that only 55% of postmenopausal women with hypertension who attended primary care achieved their BP goals, while 45% of hypertensive women, even those who took antihypertensive drugs, did not reach the desired levels. Overall, the incidence of hypertension was 32.2% in men and 30.5% in women, according to an examination of the National Health and Nutrition Survey (NHANES) (2003–2004) data on hypertension prevalence, treatment, and control. Men and women both had similar treatment rates (69.9% vs. 67.2%), but women's control rates (49.4% vs. 56.5%) tended to be lower (29). Studies indicate that two-thirds of older hypertensive women, who are at a higher risk for stroke and cardiovascular events, do not have their hypertension adequately controlled, either because they are not receiving medication or because their blood pressure remains too high despite taking antihypertensive medications (30).

A statistically higher number of patients reached their BP goals (*P* < 0.01) in the 60 (*n* = 154) years of age group than in the <60 years of age group, showing that the increase in age in women was associated with BP goal attainment. Both the systolic BP and diastolic BP rise with age until the age of 50–60 years. In individuals over the age of 60 years, systolic BP often tends to rise with ageing, whereas diastolic BP either remains stable or decreases. Gradual stiffening of the artery wall is the most common cause of the disconnection between systolic BP and diastolic BP, leading to an excessive increase in pulse pressure (31). A cross-sectional study examining the relative impact of menopausal status on BP levels in healthy women aged 35–60 years old revealed changes in systolic BP and diastolic BP based on the menopausal state (32).

This study found a significant association between achieving BP targets and high educational levels and employed status among hypertensive postmenopausal women. Self-management of chronic diseases is a challenging process because, once diagnosed, certain diseases require lifelong therapy and observation. Successful blood pressure control relies on patient and provider education. Among Korean women, the impact of education and socioeconomic status on hypertension was more pronounced compared to men (33).

Age at menopause, age at menarche, and number of children showed no significant association with BP goal attainment among postmenopausal women with hypertension. A Mendelian randomisation (MR) study of 4,451 postmenopausal women reported that early menopause was correlated with reduced DBP (*β* = 1.31, 95% CI: 2.43–0.18) and that a 1-year delay at the beginning of menopause was associated with a 2% (95% CI: 0–4) increased risk of hypertension. After adjusting for high salt intake, older women who experienced menopause later than the average age (50 years) had a 1.597-times increased risk of developing hypertension compared to those with an earlier cessation of menstruation (34, 35).

The risk of cardiovascular disease in women can be associated with age at menarche (36). According to a Chinese study, the average age at menarche was 15.5 years. Menarche age was associated with an increased risk of hypertension by 15% for each additional year (OR = 1.15, 95% CI: 1.11–1.19). There was a substantial positive correlation between age at menarche and SBP (*β* = 0.88, 95% CI: 0.29–1.46) and DBP (*β* = 0.80, 95% CI: 0.47–1.13) in women with hypertension. Younger participants (15–44 years: OR = 1.37, 95% CI: 1.21–1.55; *P* for interaction = 0.009) had a significantly higher risk of hypertension than those between 62 and 97 years of age (37). However, the present study revealed no significant association between age at menarche and BP goal attainment.

A woman's BP may vary depending on the number of pregnancies she has experienced due to physiological changes in blood perfusion during pregnancy. Increases in weight, identified as a major risk factor for hypertension, may be associated with increasing parity (38). One possible mechanism for the BP-lowering effects is an extended lifespan of breastfeeding. The extended release of oxytocin during lactation has been linked to both short-term reductions in blood pressure and a decreased risk of hypertension and CVD in middle age (39).

This study found a substantial correlation between BP target attainment and weight, with 81.3% of patients with normal weight reaching their targets compared to 46.3% of those who were overweight. These findings align with earlier studies, indicating that postmenopausal women with BMI values of 25–28 and 29 had a higher risk of having hypertension by 1.7 times (95% CI: 1.6–1.8) and 2.7 times (95% CI: 2.5–2.9), respectively, than postmenopausal women with a BMI of 25. Another study conducted on postmenopausal women in Italy found that menopausal women with BMI values between 24 and 26 had 1.48 times (95% CI: 1.39–1.57) higher risk of developing hypertension than those with normal BMI values and that those with BMI values above 26 had 2.56 times (95% CI: 2.41–2.71) higher risk of developing hypertension (40). Even among postmenopausal women without associated conditions, being overweight or obese significantly lowered mortality rates, showing that establishing BMI cutoffs for obesity management should take menopausal status into account in addition to age (41).

According to this study, 60.9% of patients received monotherapy and 39.1% received combination therapy including two or more antihypertensive drugs. Compared to other antihypertensive medications, angiotensin receptor blockers (ARBs) (56.5%) demonstrated higher rates of target achievement among hypertensive postmenopausal women. According to a study conducted in Punjab, monotherapy represented 21.82% of prescriptions, with ARBs accounting for 10% of these, and two-drug therapies, including ARB + BBs (20.91%), represented 44.09% of prescriptions. The most frequently prescribed fixed drug combination (FDC) was ARB + DI (9.55%). Three, four, and five medication combinations like ARB + BB + DI (10.45%), ARB + 2DI + BB (4.09%), and ARB + 2DI + BB + calcium channel blocker (1.82%) were recommended most frequently (42). The results of another Indian study revealed that monotherapy included ARB, accounting for 24.8% of the total prescriptions, followed by calcium channel blockers (19.4%), ACEIs (11%), beta-blockers (2.8%), and diuretics (2%). Amlodipine was the most commonly recommended medication, with 16.4% and 31.6% of patients receiving two combinations (43).

The effects of several antihypertensive medications on postmenopausal mild-to-moderate hypertension were examined in a meta-analysis. Compared to placebo, angiotensin receptor blockers were found to significantly reduce systolic blood pressure (standardised mean difference = 9.25, 95% confidence interval 12.24–6.26) (44). This study found that 40% of patients did not reach their BP goals for either therapy, which suggests that older women have stiffer arteries, which could lead to earlier pulse wave reflection and increased SBP and pulse pressure. The early return of the reflected pulse wave in late systole results from increased arterial stiffness and pulse wave velocity (PWV), which increases central pulse pressure, myocardial oxygen demand, and left ventricular workload. Hypertension and increased pulse pressure serve as two clinical indicators of decreased arterial distensibility (45, 46).

Women who do not have adequate blood pressure management may have a wide range of reasons, such as resistance to treatment and a history of conditions like diabetes or chronic renal disease, which are strongly associated with poorer blood pressure control. The number of antihypertensive medication classes used was unrelated to blood pressure control among treated hypertensive women, although diabetes and advanced age were related to poor blood pressure management (29). According to long-term studies on antihypertensive drugs, monotherapy typically reduces the risk of CVD in a significant number of patients while maintaining a satisfactory safety profile. Selecting the appropriate monotherapy for various patient types could be a primary challenge. Selecting a medication that has the most potential to lower BP while having the fewest side effects on a particular patient is often the most prudent approach (47). It has recently been clear that many postmenopausal hypertensive women do not have adequate blood pressure control when taking traditional antihypertensive drugs. To provide the best treatment options for these women, a thorough understanding of the intricate pathophysiology of postmenopausal hypertension is required (48). In hypertensive patients, the goal of BP reduces the risk of stroke by 40% and myocardial infarction by 15% (49).

Multivariate logistic regression analysis showed that poor blood pressure was not associated with age at menopause, number of children, employment status, level of education, BMI, and number of co-morbidities but was associated with age at menarche. However, studies have shown that gender-specific risk factors, such as early menarche, the age at the first childbirth, women's empowerment, and the number of births, must be considered with well-established modifiable risk factors for non-communicable diseases such as hypertension (50). The current study demonstrated that biosocial factors are not associated with BP level control among hypertensive postmenopausal women. Targeted and/or population-based methods can be implemented to prevent and control hypertension (51).

### 4.1 Strengths and limitations

The main strength of this current study is probably that it is likely to be the first study to evaluate the association between biosocial predictors and BP goal achievements among postmenopausal women in south India. However, it has some limitations, which include a small scope, sample size, shorter duration of the study period, and that it has not studied cardiovascular disease. It is recommended that future studies of a larger sample size with cardiovascular disease in postmenopausal women should be conducted to better understand the scope of the problem.

## 5 Conclusion

Hypertension is a major health problem in postmenopausal women, which is not always well controlled. Biosocial predictors are not associated with the achievement of BP goals among postmenopausal women, and half of the patients do not attain their BP goals. Approaches like lifestyle change, treatment adherence, and continuous BP monitoring will be required to expand screening coverage. Under the direction of medical professionals, plans to improve hypertension control and knowledge of the condition in women should be made.

## Data Availability

The raw data supporting the conclusions of this article will be made available by the authors without undue reservation.
